# The Role of PPAR and Its Cross-Talk with CAR and LXR in Obesity and Atherosclerosis

**DOI:** 10.3390/ijms19041260

**Published:** 2018-04-23

**Authors:** Pengfei Xu, Yonggong Zhai, Jing Wang

**Affiliations:** 1 Beijing Key Laboratory of Gene Resource and Molecular Development, College of Life Sciences, Beijing Normal University, Beijing 100875, China; pex9@pitt.edu; 2 Key Laboratory for Cell Proliferation and Regulation Biology of State Education Ministry, College of Life Sciences, Beijing Normal University, Beijing 100875, China; 3 Department of Biology Science and Technology, Baotou Teacher’s College, Baotou 014030, China

**Keywords:** PPAR, CAR, LXR, obesity, atherosclerosis

## Abstract

The prevalence of obesity and atherosclerosis has substantially increased worldwide over the past several decades. Peroxisome proliferator-activated receptors (PPARs), as fatty acids sensors, have been therapeutic targets in several human lipid metabolic diseases, such as obesity, atherosclerosis, diabetes, hyperlipidaemia, and non-alcoholic fatty liver disease. Constitutive androstane receptor (CAR) and liver X receptors (LXRs) were also reported as potential therapeutic targets for the treatment of obesity and atherosclerosis, respectively. Further clarification of the internal relationships between these three lipid metabolic nuclear receptors is necessary to enable drug discovery. In this review, we mainly summarized the cross-talk of PPARs-CAR in obesity and PPARs-LXRs in atherosclerosis.

## 1. Introduction

Obesity is a lipid metabolic disturbance that has been growing across the world for nearly half a century. It is a global human health concern. In 2016, more than 1.9 billion adults (≥18 years old) were overweight and, of these, over 650 million were obese. Furthermore, 340 million children and adolescents (5–18 years old) and 41 million children (≤5 years old) were overweight or obese [1,2]. The body mass index (BMI), defined as a person’s weight in kilograms divided by the square of their height in meters, is a simple index used to classify overweight and obesity in adults. Obesity is associated with various metabolic disorders and cardiovascular diseases. A high BMI is considered to be an indicator of high body fatness that may lead to a high risk of cardiometabolic syndrome and atherosclerotic vascular disease [3,4,5]. Atherosclerosis, also known as arteriosclerosis, hardening of the arteries, is a disease in which fatty plaque deposits build up inside the arteries, narrowing them, leading to some serious problems, including coronary artery disease, stroke, or even death [6]. Obesity and atherosclerosis are common chronic lipid metabolic disorder diseases. The treatment and prevention of obesity and atherosclerosis are both major challenges, and studying this problem can help us live longer, healthier lives.

**Figure 1:**
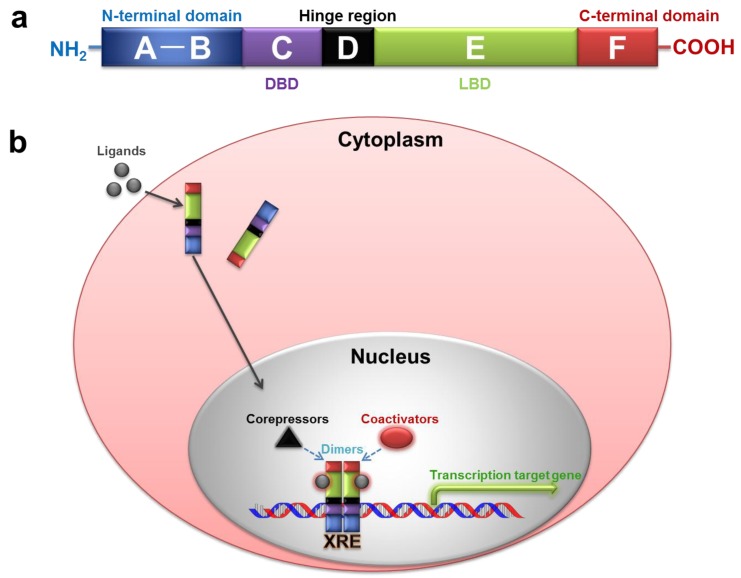
Schematic structure of NRs (nuclear receptors) and model of NR signalling. (**a**) General domain structure of NRs; and (**b**) the mechanism of general NR action. The ligands bind to the LBD (ligand-binding domain) of NRs in the cytoplasm, and translocate to the nucleus. Then the DBD (DNA-binding domain) of NRs bind to the XRE (xenobiotic responsive elements) forming dimeric complexes with RXR and the recruitment of co-activators or co-repressors. Finally, this leads to the transcription of the target genes. This model is applied to type II NRs, including PPARs, CAR, LXRs, and others. The colorful words just match the corresponding colorful shape. The dotted arrows mean different ligands can recruit coactivators or corepressors to form dimers, respectively.

Nuclear receptors (NRs), a class of ligand-activated transcriptional factors, play significant roles in metabolic homeostasis. It is well known that there are 48 and 49 NR genes in humans (*Homo sapiens*) and mice (*Mus musculus*), respectively [7,8]. Most of the NRs contain six functional domains, such as the variable N-terminal regulatory domain (A–B), the conserved DNA-binding domain (DBD) (C), the variable hinge region (D), the conserved ligand binding domain (LBD) (E), and the variable C-terminal domain (F) (Figure 1a) [7,9]. The classical function of NRs is to transcriptionally regulate the expression of cognate target genes through the recruitment of coactivators or corepressors when ligands bind to the receptors [10,11] (Figure 1b). To perform the transcriptional activity, NRs either (1) act as monomers; (2) need to form dimeric complexes (homodimers); or (3) form complexes with the retinoid X receptor (RXR) (heterodimers) and bind to the DNA in the cell nucleus [9]. Recently, many studies have indicated the role of some NRs in the regulation of lipid metabolism. It has been recognized that peroxisome proliferator-activated receptors (PPARs) act as fatty acid sensors, regulating the multiple pathways involved in lipid and glucose metabolism and overall energy metabolism [12,13]. Furthermore, the constitutive androstane receptor (CAR), which was initially characterized as a xenosensor that controls xenobiotic responses, has been recently identified as a therapeutic target for obesity and its related metabolic disorders [14,15], whereas liver X receptors (LXRs) are sterol sensors that mainly regulate cholesterol, fatty acid and glucose homeostasis, they can inhibit atherosclerosis development, but promote lipogenesis in liver [16]. In this review, we briefly summarize the roles of PPARs, CAR and LXRs and their ligands in the treatment of metabolic diseases, obesity and atherosclerosis, and discuss the cross-talk of PPARs-CAR and PPARs-LXRs in lipid metabolism regulation.

## 2. The Initial Characterization of PPAR, CAR, and LXR

### 2.1. Fatty Acids Sensor PPARs

PPARs are molecular sensors of fatty acids and fatty acid derivatives and control energy homeostasis (carbohydrate, lipid, and protein) [17]. There are three types of PPARs which have been identified: PPARα (NR1C1, encoded by *PPARA*), PPARβ/δ (NR1C2, encoded by *PPARD*), and PPARγ (NR1C3, encoded by *PPARG*). They are all lipid sensors that transcriptionally regulate diverse aspects in response to nutritional inputs, and serving as effective therapeutic targets for some types of lipid metabolic syndrome, including obesity, atherosclerosis, dyslipidaemia, type 2 diabetes mellitus (T2DM), and nonalcoholic fatty liver disease (NAFLD) [12,18]. PPARα is highly active in liver, brown adipose tissue (BAT), kidney, heart, and muscle tissue [19], where it regulates the adaptive response to prolonged fasting by controlling the process of ketogenesis, fatty acid transport, fatty acid binding, fatty acid activation and mitochondrial fatty acid β-oxidation [20,21]. Genomic studies have indicated that PPARα, as a master regulator of lipid metabolism, has various target genes; the classical genes include acyl-CoA oxidase, thiolase, fatty acid transport protein (*FATP*), carnitine palmitoyltransferase I (*CPT1*), and peroxisome proliferator-activated receptor gamma coactivator 1-alpha (*PGC-1α*) [20,22]. The expression of PPARβ/δ is highest in adipose tissue, skeletal muscle, macrophages, brain, and skin, but is at low levels in the liver, where it mainly regulates fatty acid catabolism and the glycolytic-to-oxidative muscle fibre-type switching used in improving lipid homeostasis [23,24,25,26]. PPARα and PPARβ/δ have been shown to block lipid absorption by upregulating L-type fatty acid binding protein (L-FABP) and cluster of differentiation 36 (CD36) in the small intestine [27]. PPARγ function has mainly been characterized in adipose tissue, macrophages and the colon, and it has three forms: PPARγ1, PPARγ2, and PPARγ3 through alternative splicing [28,29,30]. PPARγ1 and PPARγ3 encode the same protein, and PPARγ3 is a splicing variant of PPARγ1. PPARγ2 has 28 additional amino acids at the variable N-terminal regulatory domain compared with PPARγ1 [31]. Furthermore, PPARγ1 has been found in nearly all tissues, except muscle, whereas PPARγ2 is mostly found in the adipose tissue and intestine, and PPARγ3 is mainly expressed white adipose tissue, colon, and macrophages [32]. PPARγ was initially known as an inducer during adipocyte differentiation [33,34], and its most famous role is in regulating lipogenic pathways. Genomic studies have revealed that PPARγ controls the expression of the early adipogenic differentiation factors CCAAT-enhancer-binding proteins (C/EBPs) and fatty acid binding protein 4 (FABP4), glucose homeostasis factors glucose transporter type 4 (GLUT4), and catabolite activator protein (*CAP*) genes. Moreover, PPARγ regulates some insulin sensitive adipokines, such as leptin, adiponectin, and tumour necrosis factor α (TNF-α) [35,36,37]. PPARγ is also involved in the metabolism of long-chain unsaturated fatty acid in the intestinal epithelium [38]. Although there are many similarities in lipid and glucose homeostasis, each of the PPAR isoforms has unique functions in vivo, probably due to their differential tissue distributions, the distinct ligands, and the inherent differences in biochemical characteristics [39].

Many endogenous agonists of PPARs have been identified, including polyunsaturated fatty acids, branched chain fatty acids, nitro/oxidized-fatty acids, phospholipids, eicosanoids, prostaglandin, oleoylethanolamide, carbaprostacyclin, 5HT metabolites, and so on [40,41,42,43]. In addition, many natural and synthetic PPAR ligands have been applied to treat lipid and glucose metabolic syndrome in pharmaceutical companies, as shown in Table 1. Fibrate drugs (including bezafibrate, clofibrate, fenofibrate, gemfibrozil, ronifibrate, etc.) are a class of classical PPARα agonists used to treat hyperlipidaemia and increase high-density lipoprotein cholesterol (HDL-c) in clinical settings. Moreover, pemafibrate [44] (approved in Japan in July 2017) and LY518674 [45] (phase II) are selective PPARα modulators used as anti-atherosclerosis agents in clinical trials. PPARβ/δ agonists are currently not used in clinical applications, but seladelpar (MBX-8025) is currently a promising activator for improving mixed dyslipidaemia and normalizing alkaline phosphatase levels, and is in phase 2 clinical development [46]. Additionally, KD-3010 is also a promising PPARβ/δ agonist for the potential treatment of diabetes and obesity in the phase I clinical trial. It shows the protective and anti-fibrotic effects in liver injury induced by carbon tetrachloride (CCl_4_) injection or bile duct ligation (BDL) [47]. Thiazolidinediones (generically marked as pioglitazone, rosiglitazone, and lobeglitazone) are potent agonists of PPARγ with powerful insulin sensitizing activity which can be used in the treatment of T2DM. However, they have some undesirable side effects, such as weight gain, osteoporosis, and congestive heart failure [39,48]. Some failed and non-marked thiazolidinediones include troglitazone (marked as Rezulin, which was withdrawn due to adverse liver effects), balaglitazone, ciglitazone, darglitazone, netoglitazone, and rivoglitazone, etc. Recently, several partial agonists of PPARγ have been reported to keep beneficial antidiabetic characteristics with few side effects. Honokiol is a natural compound purified from the bark of *Magnolia officinalis* in traditional Chinese medicine, which has been identified as a novel non-adipogenic partial PPAPγ ligand. It has an anti-hyperglycemic property but does not trigger adipogenesis in vitro and in vivo [48]. Amorfrutins, as selective PPARγ modulators, are also natural products derived from two legumes, *Glycyrrhiza foetida* and *Amorpha fruticose*. They were reported to improve insulin sensitivity and dyslipidemia and protect liver steatosis without a concomitant increase of body weight gain in diet-induced obese and db/db mice [49,50]. In our recent study, Danshensu Bingpian Zhi (DBZ) is a synthetic derivative of the natural compounds *Danshensu* (*tanshinol*) and *Bingpian* (*borneol*), which are used as “sovereign” and “courier” in the traditional Chinese medicine formula Fufang Danshen (FFDS). We found that DBZ is a putative PPARγ partial activator capable of preventing insulin resistance, obesity, and atherosclerosis in mice without significant unwanted effects [51,52]. Along with improving our understanding of the biological roles of PPARs, we suggest that further study of the selectively pleiotropic PPAR agonist is a promising approach for developing further therapies.

### 2.2. Xenobiotic Receptor CAR

CAR is a member of the NR1I3 family of nuclear receptors, initially serves as a xenobiotic nuclear receptor, responding to xenobiotics and drug stress [53,54]. Androstenol, and some isomers of androstanol, androstanes, have been found to be endogenous antagonists of CAR, and dehydroepiandrosterone (DHEA), also an androstane, is an endogenous agonist of CAR. Androstanes, despite acting as ligands, are the basis for the naming of this receptor. The name “constitutive androstane receptor” refers to the unusual, constitutively-active status of this receptor when not occupied by a ligand. CAR is primarily expressed in the liver and small intestine, but is also found in the kidney, heart, and brain [55], and we also detected it in the mammary gland, ovary, and uterus (our unpublished data). It, often along with the pregnane X receptor (PXR) and vitamin D receptor (VDR), regulates the phase I and II xenobiotic metabolizing enzymes (including cytochrome P450s, sulfotransferases, glutathione-*S*-transferases) and other multidrug-resistance associated proteins used to both modulate drug metabolism and bilirubin clearance and prevent hepatotoxicity [56,57,58]. More recently, CAR has been reported to regulate both lipid and glucose metabolism and has been a potential therapeutic target for several metabolic diseases, such as obesity [15,59], atherosclerosis [60,61], NAFLD [62,63], and T2DM [64,65], due to its ability to balance the endogenous homeostasis of components, including glucose, steroids, bile acids, bilirubin, and thyroid hormone.

Since CAR has a large hydrophobic LBD pocket, a variety of chemical xenobiotics can activate it, such as clinical drugs, insecticides, flavonoids, terpenoids, polyphenols, environmental chemicals, and others [66,67]. Interestingly, CAR exhibits arresting species specificity in the ligand binding recognition between human and rodent, though both species use the same DNA response element sequences to recruit CAR. For example, TCPOBOP (1,4-*bis*[2-(3,5-dichloropyridyloxy)]benzene), is a potent mouse CAR (mCAR) agonist which only activates mouse, but not human, CAR, whereas CITCO (6-(4-chlorophenyl) imidazo [2,1-β] [1,3] thiazole-5-carbaldehyde-*O*-(3,4-dichlorobenzyl) oxime) is only a human CAR (hCAR) agonist, having no effect on mouse CAR [68,69]. Thus, this specificity should be considered when choosing the animal model for studying pharmacologic effects or drug screens targeting CAR. Phenobarbital, also known as phenobarb or phenobarbitone, is the preferred antiepileptic and sedation medicine used clinically, and it can activate both human and mouse CAR. Some early studies have shown that phenobarbital can regulate energy mentalism and improve insulin sensitivity and hepatic lipid homeostasis in ob/ob mice and human patients [70,71,72]. Activation of CAR reduced sterol regulatory element-binding protein 1 (SREBP-1) levels by inducing the expression of insulin induced gene 1 protein (*INSIG-1*), a protein blocking the proteolytic activation of SREBPs [73]. In a previous study, we reported that activation of CAR inhibited lipogenesis by suppressing LXR ligand-responded recruitment of LXR to the LXR response element (LXRE) and the expression of LXR target genes, whereas activation of LXR inhibited the CAR ligand-induced recruitment of CAR to Cyp2b10 [74]. Although CAR is a potential therapeutic target for lipid metabolic disease, some barriers exist for the clinical use of its agonists: there are concerns around hepatic enlargement and carcinogenesis. CAR also interacts with PPAR and LXR in regulating lipid and glucose homeostasis. Better understanding of these mechanistic properties might help us overcome these barriers in the future.

### 2.3. Oxysterol Sensor LXRs

LXRs are well-known nuclear oxysterol receptors that have two isotypes: LXRα (NR1H3) and LXRβ (NR1H2). LXRα is highly active in the liver, intestines, kidneys, adipose tissue, lungs, macrophages, and adrenal glands. LXRβ, also named as a ubiquitous receptor, is expressed in almost all tissues and organs [75,76,77]. Both of them may control cholesterol, fatty acid, and glucose metabolism to protect against atherosclerosis, lipid disorders, diabetes, chronic inflammation, Alzheimer’s disease, and even cancer [78,79,80,81].

In cholesterol and lipid homeostasis, activation of LXR can stimulate reverse cholesterol transport and reduce the body’s cholesterol overload by inducing the sterol metabolism and transporter network, including cytochrome P450 family 7 subfamily A member 1 (CYP7A1), ATP-binding cassette sub-family A member 1 (ABCA1), ABCG1, ABCG5, ABCG8, and apolipoprotein E (ApoE) [82,83,84]. Furthermore, LXR activation also results in an increase in lipid synthesis in the liver through inducing the expression of SREBP-1c, fatty acid synthase (FAS), acetyl-CoA carboxylase 1 (ACC1), and stearoyl-CoA desaturase 1 (SCD-1) [85,86,87]. LXRs, as sterol sensors, have a variety of endogenous activators, most of which are oxidation products of cholesterol, such as 27-hydroxycholesterol, 22(*R*)-hydroxycholesterol, 20(*S*)-hydroxycholesterol, 24(*S*)-hydroxycholesterol and 24(*S*), and 25-epoxycholesterol [16,76,88]. Interestingly, these endogenous agonists, unlike natural synthetic LXR activators, do not activate the SREBP signal pathway [89,90,91]. Several studies have reported that mice treated with synthetic LXR activators, including GW3965 and TO901317, show enhanced hepatic and serous triglyceride levels, and have promoted very low-density lipoprotein (VLDL) secretion [86,92,93]. These shortcomings limit the use of LXR activators in clinical settings. LXRα is the major sensor of dietary cholesterol. Mice lacking LXRα cannot induce transcription of the gene encoding cholesterol 7α-hydroxylase (CYP7A), which is a rate-limiting enzyme in bile acid synthesis. LXRα^−/−^ mice are healthy when fed with a normal chow (low cholesterol) diet. However, they develop enlarged fatty livers with high cholesterol levels, and lead to impaired hepatic function when fed a high-cholesterol diet [94]. LXR-623 (WAY-252623) is the first LXRα-partial/LXRβ-full agonist used for the treatment of atherosclerosis in animal models and has been tested in a phase I clinical trial. However, the trial was terminated due to adverse effects on the central nervous system [95,96]. Similar synthetic agonists, including CS8080, BMS-852927 (also named XL-041) have been terminated for undisclosed reasons, and only BMS-779788 (also named XL-652) has proved safe enough to continue with clinical trials [97,98], the detailed information as shown in Table 2. LXR activators can reduce cholesterol level in blood and liver. They also improve glucose tolerance in mice by decreasing insulin resistance. Human functional and genetic analysis showed that the common LXR promoter SNPs rs35463555 and rs17373080 may regulate sensibility to T2D [99]. We recently reported that DBZ inhibits foam cell formation and protects against atherosclerosis in ApoE^−/−^ mice through activating LXRs [52,100]. DBZ also activates PPARγ and prevents high fat diet-induced obesity, insulin resistance and gut dysbiosis in mice [51]. By clarifying the cross-talk between PPARs and LXRs we may gain a better understanding of their synactic function in cholesterol and lipid homeostasis.

## 3. Cross-Talk of PPARs and CAR Links to Obesity

PPARs and CAR are both essential lipid metabolic nuclear receptors active in controlling obesity and its related metabolic disorders. PPARs are quite interesting. PPARα and PPARβ/δ are potential targets to prevent obesity [101,102,103], by the mechanism as mentioned above in Section 2.1. Contrarily, PPARγ is a master regulator of adipocyte differentiation both in vivo and in vitro [104]. A lack of PPARγ results in the inability to develop adipose tissue, as seen in PPARγ knockout mice [105,106]. Thiazolidinediones, as famous PPARγ activators, are a group of anti-diabetic drugs to treat T2MD, but can lead to serious side effects. Weight gain is an unwanted side effect: activation of PPARγ in adipose tissue stimulates the expression of genes leading to lipogenesis, including *AP2*, *CD36*, *SCD-1*, *SREBP-1*, and others, which promote lipid storage [18]. PPARα, as a key nutritional sensor, regulates the metabolism of lipids, carbohydrates, and amino acids [107]. It is a potential therapeutic target for the treatment of obesity, hypertriglyceridemia, NAFLD, and atherogenic dyslipidaemia [108,109,110]. Oestrogen inhibits the actions of PPARα on obesity and lipid metabolism through its effects on the PPARα-dependent regulation of target genes [111,112]. CAR, as a therapeutic target for obesity, was reported about ten years ago. Activation of CAR also increased faecal bile acid excretion and attenuated atherosclerosis in low-density lipoprotein receptor-deficient (LDLR^−/−^) and ApoE^−/−^ mice by increasing reverse cholesterol transport [60,61]. Recently, we reported that activation of CAR with TCPOBOP inhibited lipogenesis and promoted fibrosis in the mammary gland of adolescent female mice [113]. The classical CAR agonist TCPOBOP has a robust anti-obesity phenotype in high-fat diet-induced obese mouse models. Mechanically, activation of CAR improves insulin sensitivity, inhibits lipogenesis and gluconeogenesis, and increases brown adipose tissue energy expenditure.

The cross-talk between PPARs and CAR in obesity can be achieved through their target gene PGC-1α. PGC-1α, as a transcriptional coactivator, interacts with nuclear receptor PPAR and controls energy metabolism through the regulation of mitochondrial biogenesis [114,115]. CAR regulates the degradation of PGC-1α by recruiting E3 ligase targeting PGC1α and promoting ubiquitination in the liver [116]. During fasting, the PPARα activator WY14643 induces both CAR and its target gene CYP2B expression in a PPARα-dependent manner in rat hepatocytes [117,118]. Meanwhile, Guo et al. reported that synthetic PPARα ligands ciprofibrate, clofibrate, and others drove adenoviral-enhanced green fluorescent protein-CAR into the hepatocyte nucleus in a PPARα- and PPARβ-independent manner in mouse liver in vivo. More interestingly, molecular docking assay showed that PPARα activators, Wy-14643 and ciprofibrate, could fit into the ligand binding pocket of CAR and their binding modes were similar with that of androstanol, an endogenic CAR inverse agonist. PPARα activators interfered with coactivator recruitment to the LBD of CAR and suppressed the constitutive transactivation of CAR. Mechanistically, the transcription coactivator PPAR-binding protein (PBP) plays a pivotal role in nuclear translocation of CAR in mouse liver, but not the PPAR-interacting protein (PRIP) [119,120]. These results indicated that activation of PPARα by some ligands induced nuclear translocation of CAR. β-oxidation is also controlled by both PPARs and CAR. PPARα regulates mitochondrial fatty acid β-oxidation by inducing the gene expression of *CPT1*, as previously mentioned. Conversely, the CAR ligand pentobarbital inhibits mitochondrial CPT1 expression and β-oxidation, resulting in increasing ketone production in serum [8,121]. However, in BAT, activation of CAR by TCPOBOP significantly increased expression of *PGC-1α* and β-oxidation [15]. Hence, the cross-talk between PPAR and CAR should be separately considered for different tissue types. Above all, the dual functions of PPAR activators have possible cross-talk with CAR through target gene *PGC1α*, coactivator recruitment, and mitochondrial fatty acid β-oxidation in different conditions in energy metabolism.

## 4. Cross-Talk of PPARS and LXRS in Atherosclerosis

There is a potential cross-talk or interaction between PPARs and LXRs in the prevention and treatment of atherosclerosis. Most nuclear receptors form heterodimers with RXR, including PPAR/RXR, LXR/RXR, CAR/RXR, and others. Ide et al. has elegantly reported that LXR-RXR-PPAR forms a network that regulates fatty acid metabolism and lipid degradation [122]. These compounds enhance binding to their respective target gene promoters. Unsaturated fatty acids increase the expression of LXRα, but not the LXRβ in rat liver cells, both in vivo and in vitro. This upregulated effect of LXRα is associated with the transcriptional rate and binding of PPARα to PPAR response element (PPRE). Meanwhile, a PPRE is found in the human LXRα flanking region [123]. *SREBP-1c*, as a direct target gene regulated by LXR, is crucial in both lipid and sterol biosynthesis. Luciferase assays have proven that the activation of PPARα and PPARγ reduces LXR-induced *SREBP-1c* promoter activity and gel shift assays have demonstrated that PPARs inhibit the binding of LXR/RXR to LXRE [124]. Thus, PPARs and LXRs play opposite roles in regulating triglyceride synthesis in the liver and serum. LXRα also inhibits peroxisome proliferator signalling through cross-talk with PPARα [125]. Moreover, Liduo Yue et al. reported that LXRs could bind to PPARs with different binding affinities in vitro using surface plasmon resonance technology and molecular dynamics simulation [126].

Despite the opposite roles in triglyceride homeostasis, PPARs and LXRs have some common ground in their anti-atherosclerotic effects. In foam cell macrophages, both PPARα and PPARγ (through the LXR-dependent ABC pathway) control cholesterol efflux [127,128], and activation of PPARα and PPARγ both prevent foam cell formation and atherosclerosis development in ApoE^−/−^ and LDLR^−/−^ mice [129,130]. Activation of LXRα also raises the expression of ABCA1 and ABCG1, which accelerate the reverse transport of cholesterol and then deposit in the liver [131]. PPAR-LXR-ABCA1 is an important pathway involved in cholesterol efflux and atherogenesis. In intestine tissue, the activation of LXR also increases the expression of ABCG5 and ABCG8 which regulate absorption of cholesterol and protect against atherosclerosis [79,132]. PPARs activation has performed similar acts inhibiting intestinal cholesterol absorption in rats and mice [133,134]. Taken together, both LXR and PPAR promote the movement of cholesterol from peripheral cells to the feces, which is referred to as reverse cholesterol transport (RCT). 

Atherosclerosis is a chronic inflammatory disease; inflammation plays an important role in the pathogenesis and progression of atherosclerosis [135,136]. Recent studies have revealed the mechanism by which PPARs and LXRs regulate the inflammation process through some inflammatory target genes. Activation of PPARs and LXR can inhibit lipopolysaccharide- and cytokine-induced pro-inflammatory gene expression by repressing the toll-like receptor (TLR)-nuclear factor kappa B (NF-κB) signal pathway [137,138,139]. PPARα increases the expression of inhibitor of kappa B (IκB) to antagonize the NF-κB signalling pathway [140]. PPARβ/δ induces transforming growth factor beta (TGF-β) and inhibits the activation of NF-κB, thus regulating inflammatory processes [141]. Thiazolidinediones (TZDs) induced PPARγ activation also reduced the expression of inflammatory factors, including TNF-α and gelatinase B, in the aortic root, thus inhibiting the development of atherosclerosis [142]. All three PPAR isoforms regulate the immune response through different cell-signalling systems. LXRs repress inflammatory pathways through their transcriptional mechanisms [143,144]. LXRs and PPARγ control immunity by mediating proinflammatory gene transrepression through parallel small ubiquitin-like modifier (SUMO) ylation-dependent pathways [145]. PPARs and LXRs have been a critical interface for inflammation and cholesterol homeostasis. Concurrent activation of LXR and PPAR may have some beneficial effects. Activation of LXR by TO901317 and PPARα by fenofibrate in combination improves glucose tolerance, alleviates insulin resistance, and blocks TO901317-induced hyperlipidaemia, but aggravates hepatic steatosis in high fat diet-induced obese mice [146]. TO901317 and fenofibrate are both potent agonists. Concurrent partial agonists of LXR and PPAR may keep beneficial characteristics with few side effects. In our recently study, DBZ, as a promising therapeutic agent for atherogenesis and obesity in the mouse models, inhibits inflammation, macrophage migration, and foam cell formation, possibly through the partial activation of both PPARγ and LXRs.

## 5. Conclusions

**Figure 2:**
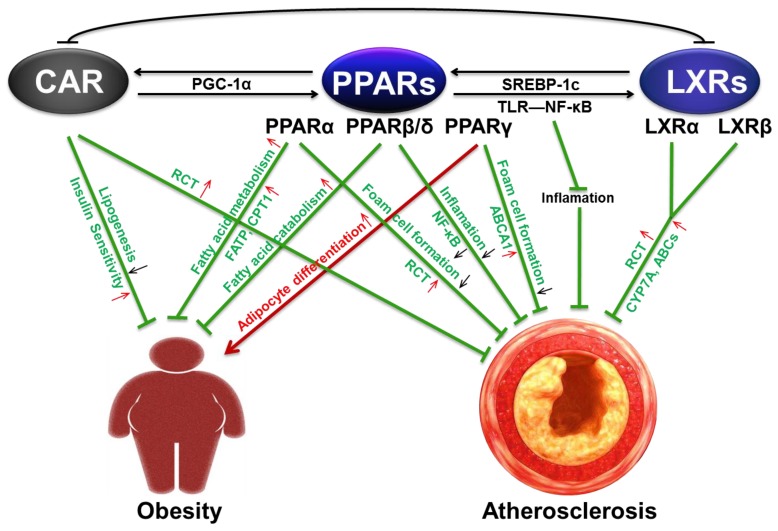
Proposed model of the cross-talks between PPARs and CAR in obesity and PPARs and LXRs in atherosclerosis. Red arrows: promotion; green T-bar: inhibition; red up-arrows: up-regulation; black down-arrows: down-regulation.

PPARs, CAR, and LXRs are a part of nuclear hormone receptors that form heterodimers with RXR to regulate lipid metabolism. Ligand binding results in DNA binding and then triggers target gene expression. Obesity and atherosclerosis are both chronic lipid metabolic disorders, which were traditionally regarded as lipid deposition diseases, principally involving triglycerides in adipose tissue and cholesterol ester in arteries. Although they are distinct conditions, obesity is often associated with atherosclerosis. Recent findings have revealed the biological roles and mechanisms of these three NRs in obesity and atherosclerosis. These receptors have been potential therapeutic targets for drug discovery; further clarification and consideration of the internal relationship between them is necessary. In this study, we summarized the interaction of PPARs and CAR in lipid metabolism and obesity-related metabolic syndrome, and the cross-talk between PPARs and LXRs in cholesterol homeostasis and atherosclerosis (Figure 2). Concurrent activation of these NRs may have some beneficial effects in lipid metabolic disease. In recently study, we reported that DBZ prevented high fat diet-induced obesity and related metabolic disorders and attenuated atherosclerosis through concurrent partial activation of both PPARγ and LXRs. Moreover, it had no apparent side effects.

Beyond these cross-talks, more NRs, such as PXR, farnesoid X receptor (FXR), aryl hydrocarbon receptor (AhR), and retinoid-related orphan receptors (RORs), are being investigated. Future studies should focus on the complex network between these NRs and how that network affects their functions. We hope that by establishing a better understanding of nuclear receptor cross-talk between metabolic disorder diseases, we can reveal promising therapeutic targets for future research.

## Figures and Tables

**Table 1:** Different PPAR ligands and their development status regarding the treatment of lipid and glucose metabolic syndrome.

Ligands	Classification	Structure	Indication	Current Stage
Bezafibrate	PPARα agonist	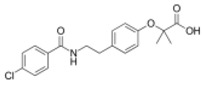	Hyperlipidemia	On the market
Clofibrate	PPARα agonist	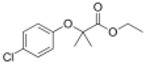	Hyperlipidemia	Discontinued
Fenofibrate	PPARα agonist	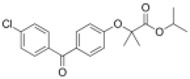	Hypercholesterolemia, mixed dyslipidemia	On the market
Gemfibrozil	PPARα agonist	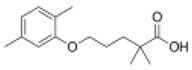	Hyperlipidemia, ischaemic disorder	On the market
Pemafibrate	PPARα agonist	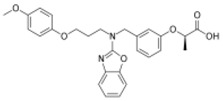	Lipid modifying agent	On the market in Japan
LY518674	PPARα agonist	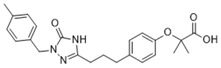	Atherosclerosis	Phase II
Seladelpar (MBX-8025)	PPARβ/δ agonist	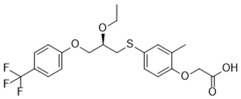	Dyslipidaemia, T2D, NASH	Phase II
KD-3010	PPARβ/δ agonist	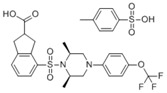	Diabetes, obesity, dyslipidemia	Phase I
Troglitazone	PPARγ agonist	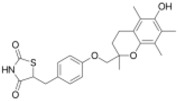	T2D	Withdrawn due to hepatotoxicity
Rosiglitazone	PPARγ agonist	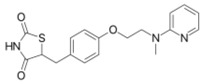	T2D	Withdrawn due to risk of CV events
Pioglitazone	PPARγ agonist	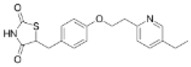	T2D	On the market
Lobeglitazone	PPARα/PPARγ agonist	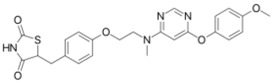	T2D	On the market in Korea
Balaglitazone (DRF-2593)	PPARγ agonist	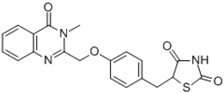	T2D	Phase III Discontinued
Ciglitazone	PPARγ agonist	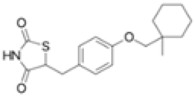	T2D	Phase II Discontinued
Darglitazone	PPARγ inhibitor	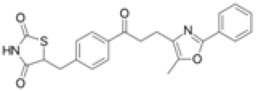	T2D	Phase I Discontinued
Netoglitazone (MCC-555)	PPARα/PPARγ agonist	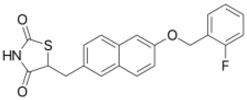	T2D	Phase II Discontinued
Rivoglitazone	PPARγ agonist	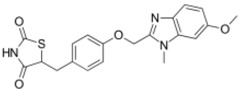	T2D	Phase III Discontinued
Honokiol	PPARγ agonist	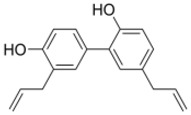	Gingival diseases, anti-hyperglycemic property	Phase III

**Table 2:** Different LXR ligands and their development status regarding anti-atherosclerosis.

Ligands	Classification	Structure	Indication	Current Stage
LXR-623 (WAY-252623)	LXRα-partial LXRβ-full agonist	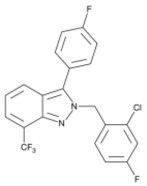	Atherosclerosis	Phase I Discontinued
BMS-852927 (XL-041)	LXR modulator	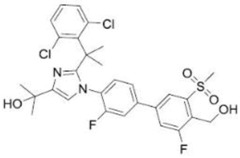	Atherosclerosis, hypercholesterolemia	Phase I Discontinued
BMS-779788 (XL-652)	LXR agonist	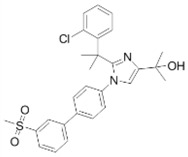	Atherosclerosis	Phase I
